# Prevalence of cardiovascular events in genetically confirmed versus unconfirmed familial hypercholesterolaemia

**DOI:** 10.21542/gcsp.2020.24

**Published:** 2020-11-30

**Authors:** Tina Z Khan, Jane Breen, Emma Neves, Richard Grocott-Mason, Mahmoud Barbir

**Affiliations:** Harefield Hospital, Royal Brompton & Harefield NHS Foundation Trust Hospital, Hill End Road, Harefield UB9 6JH, United Kingdom

## Abstract

**Introduction:** Genetic testing for familial hypercholesterolaemia (FH) is not yet established for widespread use internationally to provide diagnostic confirmation, in part due to high cost and resource requirement. We need to establish whether genetic testing is clinically justified in terms of risk stratification and prediction of cardiovascular events.

**Methods:**We performed a single tertiary cardiac centre retrospective evaluation of patients with FH managed within our genetic screening service. We evaluated the prevalence of cardiovascular events in genetically confirmed cases of FH compared to those unconfirmed upon genetic testing, to assess whether gene positivity confers a higher risk phenotype. We also compared the clinical characteristics of the genetically confirmed and unconfirmed group.

**Results:**Amongst adult patients (≥18 years) with genetically confirmed heterozygous FH (n=87), 34% (30/87) had one or more documented CV events. In comparison a lower event rate was observed in adult patients with genetically unconfirmed FH (n=170) with 25% (42/170) experiencing one or more documented CV events. Additional cardiovascular risk factors were more prevalent in the unconfirmed group including hypertension, co-morbidities, higher age and body mass index which may have modified the difference in cardiovascular risk.

**Conclusion:**Genetic testing in FH may be clinically justified and appears to identify a subset of patients with higher risk of cardiovascular events. However, the risk difference is modified by alternative cardiovascular risk factors and co-morbidities which may be more prevalent in genetically unconfirmed FH.

## Introduction

Familial hypercholesterolemia (FH) is a genetic disease characterized by elevated LDL-cholesterol (LDL-C) which deposits in blood vessels, leading to premature cardiovascular disease. It can also deposit in tissues, causing external manifestations of the disease, namely tendinous xanthomas, xanthelasmas, and corneal arcus.^1^ FH is not as rare as previously thought, affecting up to 250,000 individuals in the UK, with an estimated prevalence of 1/270 individuals in the general population with the heterozygous form.^2^ In addition, it is currently estimated that only 15 to 20% of patients with FH are actually diagnosed.^3,4^

Recently we reported on our paediatric FH genetic testing and follow-up service in which 201 children and young people between the ages of 1 and 18 years were genetically tested for FH between 2011 and 2016.^5^ Genetic testing involved 26 index cases and 175 patients were part of the cascade testing programme. In total, 110/201 or 55% of these patients were confirmed to have FH by genetic testing (57 males and 53 females); 108 were identified with heterozygous FH and two as homozygous FH, with the majority of pathogenic variants identified in the LDL receptor gene.^5^ None of the patients within this paediatric cohort had cardiovascular events either at baseline or during the follow-up period.^5^

Accurate diagnosis and identification of affected patients and timely implementation of preventative therapy is becoming increasingly relevant with the fast-paced development of novel therapies such as proprotein convertase subtilisin/kexin type 9 (PCSK9) inhibitors, which could have a major impact on reducing cardiovascular risk. A major challenge for clinicians is that there is no international consensus on diagnostic criteria, with widely varying models of diagnosis worldwide;^6^ with some countries favouring genetic screening and others relying solely on clinical criteria if at all. National UK clinical guidelines recommended genetic testing to confirm a clinical diagnosis^7^ and only a few countries, including the UK, currently have national genetic screening programs for FH.^6^

To add to the confusion, there are multiple sets of clinical criteria for the diagnosis of FH, of which three are statistically and genetically validated criteria and most commonly used: the Dutch,^8^ the Simon Broome UK,^9^ and the MEDPED US criteria.^10^

Genetic testing remains relatively expensive, costing approximately £250 for an index test;^11^ although these costs have reduced considerably in recent years^12^ due to higher volumes of testing and more cost-efficient techniques. Ultimately, we need to establish whether a test is clinically justified in terms of risk stratification and prediction of cardiovascular events. There is still limited data on the incremental clinical value of genetic screening versus clinical diagnosis alone.

Limitations also remain with genetic screening which assesses for monogenetic mutations^6^ and in many centres, cannot account for polygenic hypercholesterolaemia which may be present in the genetically unconfirmed group. In the future, causative genetic mutations beyond those currently tested may be identified.

Therefore, in our tertiary cardiac centre which has a well-established genetic screening programme for FH, we sought to establish the prevalence of cardiovascular events in genetically confirmed cases of FH compared to those unconfirmed upon genetic testing, to assess whether gene positivity confers a higher risk phenotype. We also wanted to compare the clinical characteristics of the genetically confirmed and unconfirmed group.

## Methods

This was a single tertiary cardiac centre retrospective local service evaluation or audit of patients with FH managed by the FH genetic screening and management service.

The evaluation was undertaken at our institution by retrospective review of pre-existing local FH databases, supplemented by patients’ electronic and paper medical records. Eligible patients were identified by members of the direct care team from the local FH databases and hospital patient administration systems.

It was originally intended that all identified eligible patients would be included in the evaluation; however, this was not possible within the time and data collection resource available, and data were collected for 346 of the 506 potentially eligible patients originally identified. Eighty-nine patients were subsequently excluded as they did not meet the eligibility criteria, leaving a final sample of 257 patients. Data for this service evaluation was collected covering the period 05 October 2005 to 08 April 2016.

For the purposes of this analysis, paediatric patients under the age of 18 were not included as they did not have any cardiovascular events either at baseline or during the follow-up period as we previously reported.^5^

All patients included in the study were screened for mutations in LDLR, APOB and PCSK9 using standard molecular genetic techniques.

All data were collected by members of the direct care team with no requirement for patient or next of kin consent and were recorded in pseudonymised form. For all patients included in the evaluation, data were collected on baseline demographic and disease characteristics at the time of first contact with the FH service, genetic screening results and cardiovascular outcomes during the period of follow-up with the service. Occurrence of cardiovascular (CV) events (i.e., death from CV causes, non-fatal MI, non-fatal CVA, TIA, unstable angina, CABG, PCI, PTCA and target vessel revascularisation) either prior to first contact or during the period of follow-up with the RBHT FH service were recorded. Where applicable, data on lipid parameters, management pathways and resource use for the most recent three years of management were also collected.

The collected pseudonymised data were released to pH Associates, an independent research consultancy, for data management, statistical analysis and reporting.

Approval by an NHS Research Ethics Committee (REC) was not sought since local service evaluations are exempt from REC review in the UK. However, local management approval for the evaluation’s conduct and for release of pseudonymised data to pH Associates was obtained prior to commencement of data collection.

## Eligibility criteria

The following criteria were applied to identify suitable patients for this evaluation.

### Inclusion criteria

• Patients who were genetically screened for FH.
• Patients with a diagnosis of FH (either genetically confirmed FH [heterozygous or homozygous] *or* genetic test result was inconclusive, but patient meets a modified version of the Simon Broome criteria [See Box 1] and is receiving treatment for the condition).
• Adults defined as ≥18 years at date of genetic screening.
• Patients whose routine management, following genetic screening took place at our institution under the FH service (i.e., one or more management follow-up visits).
• Patients whose first contact with the FH service was ≥12 months before the date of data collection.

### Exclusion criteria

• Patients in whom FH was deemed ‘unlikely’ based on the results of genetic testing and/or clinical presentation.
• Patients whose routine management (following genetic screening) was performed at another institution or another department within the institution outside of the FH service.
• Patients whose medical records were unavailable for retrospective review.
• Patients whose first contact with the FH service was ≤12 months before the date of data collection.

Distributions and descriptive statistics of both central tendency (medians and arithmetic or geometric means) and dispersion (standard deviation, interquartile range) are presented for quantitative variables. Nominal variables are described with frequencies and percentages, while ordinal variables also have medians and interquartile ranges described.

BOX 1 **Modified Simon Broome Criteria for defining Familial Hypercholesterolaemia**

The diagnostic criteria for familial hypercholesterolaemia (FH) using the Simon Broome register are as follows [1]:

**Definite FH is defined as:**

a) Total cholesterol >6.7 mmol/l or low-density lipoprotein (LDL) cholesterol above 4.0 mmol/l in a child <16 years or total cholesterol >7.5 mmol/l or LDL cholesterol above 4.9 mmol/l in an adult. (Levels either pre-treatment or highest on treatment)

PLUS

b) Tendon xanthomas in patient, or in first-degree relative (parent, sibling, child), or in second-degree relative (grandparent, uncle, aunt)

OR

c) DNA-based evidence of an LDL receptor mutation or familial defective apo B-100.

**Possible FH is defined as:**

a) above PLUS ONE OF d) or e)

d) Family history of myocardial infarction: below age of 50 in second-degree relative or below age 60 in first-degree relative

e) Family history of raised cholesterols: >7.5 mmol/l in adult or second-degree relative or >6.7 mmol/l in child or sibling under 16.

Our centre uses a modified version of the Simon Broome criteria for diagnosis of possible FH. Point d) (above) is replaced with “*personal or family history of cardiovascular disease: below age of 50 years in second-degree relative or below age 60 years in first-degree relative*”. This is because the term cardiovascular disease is more inclusive and may for example include ischaemic strokes which are clinically relevant; which would otherwise be overlooked using the conventional Simon Broome criteria which only includes myocardial infarction. In addition, a personal history of premature cardiovascular disease is clinically highly relevant and would potentially be overlooked using the conventional Simon Broome criteria.

**Table 1:** Patient baseline characteristics in genetically confirmed vs unconfirmed FH.

**Variable**	**Genetically Confirmed**	**Genetically Unconfirmed**
n	87	170
Age at referral (years)	48.9 (14.9)	56.7 (10.6)
Male	45 (52)	93 (55)
Female	42 (48)	77 (45)
Ethnicity: White	66 (76)	131 (77)
Asian	6 (7)	19 (11)
Black	2 (2)	3 (2)
Other	13 (15)	17(10)
Body-mass index (kg/m^2^)	26.2 (4.2)	29.5 (16.6)
Hypertension	22 (25)	105 (62)
Smoking: Current	19 (22)	25 (15)
Ex	21 (24)	44 (26)
Never	41 (47)	84 (49)
Unknown	6 (7)	17 (10)
Prior coronary artery bypass graft surgery	9 (10)	8 (5)
Prior percutaneous coronary intervention	16 (18)	28 (16)
Prior myocardial infarction	15 (17)	16 (9)
Prior stroke	2 (2)	1 (1)
Unstable angina	5 (6)	10 (6)
Co-morbidities	36 (41)	101 (59)
Atherosclerosis	6 (7)	9 (5)
Peripheral vascular disease	0 (0)	1 (1)
Hypertension	9 (10)	21 (12)
Diabetes mellitus	3 (3)	9 (5)
Liver disease	0 (0)	0 (0)
Renal disease	0 (0)	0 (0)
Pancreatic disease	0 (0)	0 (0)
Thyroid disease	6 (7)	13 (8)
Other	12 (14)	48 (28)
Lipid lowering medications		
Any Statin	54 (62)	87 (51)
Atorvastatin	26 (30)	44 (26)
Rosuvastatin	11 (13)	6 (4)
Simvastatin	14 (16)	35 (21)
Pravastatin	2 (2)	2 (1)
Ezetimibe	14 (16)	9 (5)
Bile acid sequestrant	1 (1)	0 (0)
Fibrate	2 (2)	9 (5)
Nicotinic acid	0 (0)	0 (0)
Lipids at first contact		
Total cholesterol (mmol/L)	7.2 (2.1)	6.6 (1.7)
LDL cholesterol (mmol/L)	5.2 (1.9)	4.4 (1.4)
HDL cholesterol (mmol/L)	1.3 (0.3)	1.3 (0.4)
TG cholesterol (mmol/L)	1.3 (1.0)	2.6 (3.7)
TC: LDL cholesterol ratio	5.8 (2.5)	5.1 (1.6)
Lp(a) (mg/dL)	71.6 (67.0)	59.0 (46.5)
Highest untreated lipids		
Total cholesterol (mmol/L)	9.5 (2.3)	8.6 (1.6)
LDL cholesterol (mmol/L)	6.6 (2.2)	5.9 (0.8)
HDL cholesterol (mmol/L)	1.4 (0.4)	1.4 (0.4)
TG cholesterol (mmol/L)	1.6 (0.9)	3.6 (2.1)
TC: LDL cholesterol ratio	6.9 (2.8)	6.1 (1.8)
Lp(a) (mg/dL)	69.7 (16.2, 116.8)	42.2 (12.9, 100.9)

**Notes.**

Data are mean (SD), n (%), median (interquartile range). LDL = low-density lipoprotein, HDL=high-density lipoprotein, TG=Triglyceride.

## Results

Amongst the 257 adult patients (defined as ≥18 years at date of genetic screening) included in the study, 34% or 87/257 had genetically confirmed heterozygous FH (corresponding to “definite FH” using the Simon Broome criteria); of which 94% had the LDLR mutation (82/87), 5% (4/87) had the ABOB mutation and only 1% (1/87) had the PCSK9 mutation; consistent with the mutation distribution observed in earlier studies.^13,14^ The genetically unconfirmed group (n=170) corresponded to “possible FH” using the Simon Broome criteria). In the genetically confirmed heterozygous FH group (n=87), 34% (30/87) had one or more documented CV events either prior to first contact or during the period of follow-up with the RBHT FH service. In comparison a lower event rate was observed in adult patients with genetically unconfirmed FH (n=170) with 25% (42/170) experiencing one or more documented CV events.

The most common CV events occurring in the follow-up period were PCI/PTCA (a total of 42 events, occurring a mean 6.5 years after the first contact), unstable angina (eight events, occurring a mean 2.9 years after the first contact), CABG (seven events, occurring a mean 2.6 years after the first contact), non-fatal MI (five events, occurring a mean 6.3 years after the first contact) and target vessel vascularization (five events, occurring a mean 1.3 years after the first contact).

During the follow-up period, there was a total of 26 events in those with genetically confirmed heterozygous FH, occurring a mean of 6.2 years after the first contact; and 55 events in those with genetically unconfirmed FH, occurring a mean of 4.8 years after the first contact. The most common CV event occurring in the follow-up period was PCI or PTCA, with a total of 14 events in the genetically confirmed group and 28 events in the genetically unconfirmed group.

Table 1 demonstrates baseline characteristics of the genetically confirmed compared to the unconfirmed group. There are some notable differences between the groups. The mean age at referral (years) of the genetically confirmed group 48.9 (14.9) is lower than the genetically unconfirmed group 56.7 (10.6). The male to female ratio is similar between both groups. The mean body-mass index (kg/m^2^) of the genetically confirmed group 26.2 (4.2) is lower than the genetically unconfirmed group 29.5 (16.6). The presence of hypertension is significantly less prevalent in the genetically confirmed group (25%) than in the genetically unconfirmed group (62%). History of prior coronary artery bypass graft surgery is more common in the genetically confirmed compared to the unconfirmed group (10% versus 5%), along with history of previous myocardial infarction (17% versus 9%). However, there were no significant differences in the history of previous percutaneous coronary intervention, stroke or unstable angina. Overall, the presence of co-morbidities is less prevalent in the genetically confirmed group (41%) compared to the unconfirmed group (59%). As expected, a higher proportion of the genetically confirmed group are on Statin therapy (62% versus 51%) and treatment with Ezetimibe (16% versus 5%). The lipid profiles at first contact as well as the highest untreated lipids were both less favourable in the genetically confirmed group compared to the unconfirmed group. Specifically, the mean highest untreated total cholesterol (mmol/L) was 9.5 (2.3) in the confirmed group and 8.6 (1.6) in the unconfirmed group. The mean highest untreated LDL cholesterol (mmol/L) was 6.6 (2.2) in the confirmed group and 5.9 (0.8) in the unconfirmed group. The mean HDL cholesterol (mmol/L) 1.4 (0.4) was the same in both groups. The mean highest untreated TG cholesterol (mmol/L) was 1.6 (0.9) in the confirmed group and 3.6 (2.1) in the unconfirmed group. The mean highest untreated TC: LDL cholesterol ratio was 6.9 (2.8) in the confirmed group and 6.1 (1.8) in the unconfirmed group. Lastly, the mean highest untreated Lp(a) (mg/dL) was 69.7 (16.2, 116.8) in the confirmed group and 42.2 (12.9, 100.9) in the unconfirmed group.

## Discussion

This single tertiary cardiac centre retrospective evaluation of patients with FH has demonstrated a higher prevalence of cardiovascular events in patients with genetically confirmed heterozygous FH (n=87), 34% (30/87); as compared to patients with genetically unconfirmed FH (n=170) with a prevalence of 25% (42/170). As anticipated, the data shows that genotype positivity confers a higher cardiovascular risk profile. However, the relatively high prevalence of cardiovascular events in the genetically unconfirmed group serves to highlight the fact that the cardiovascular risk in this group remains significant. Differences in the patient baseline characteristics may help to explain this. There was a higher prevalence of alternative cardiovascular risk factors in the unconfirmed group. Specifically, the mean age and body-mass index of the genetically unconfirmed group was higher (Table 1). In addition, the presence of hypertension and any co-morbidity was significantly more prevalent in the genetically unconfirmed group. It is worth specifically noting that the mean age of the genetically confirmed FH group was 48.9 years or 8 years younger than the mean age of 56.7 years in the unconfirmed group, implying that cardiovascular events tend to occur at a younger age for the genetically confirmed group suggesting a higher risk profile which has also been observed in other studies.^15^ Potentially a larger sample size may have allowed risk-adjustment for age which may have exposed an even greater increase in events in the genetically confirmed group.

As we may expect, lipid profiles in terms of both lipids at first contact as well as highest untreated lipids are worse in the genetically confirmed group (Table 1). Accordingly, the genetically confirmed group have a higher requirement for lipid lowering therapy such as statins and ezetimibe (Table 1); which may in turn mitigate the cardiovascular risk difference.

These differences in characteristics between the two groups are entirely consistent with recent data from the UK Simon Broome FH register which also demonstrated that patients with genetically unconfirmed FH had significantly higher blood pressure, BMI, prevalence of diabetes and significantly lower total and LDL cholesterol levels.^15^

There was a higher Lp(a) level in the genetically confirmed group. A recent large cross-sectional analysis demonstrated that patients with genetically confirmed FH, especially those with CVD, had higher Lp(a) plasma levels compared with their unaffected relatives (p < 0.001).^16^ Patients carrying null mutations and Lp(a) levels >50 mg/dl showed the highest cardiovascular risk compared with patients carrying the same mutations and Lp(a) levels <50 mg/dl.^16^ Therefore, higher levels of Lp(a) amongst those with genetically confirmed FH may be one of the drivers of increased cardiovascular risk.

Interestingly the mean baseline and highest untreated triglyceride cholesterol levels (mmol/L) were higher amongst the genetically unconfirmed group (2.6 and 3.6 respectively) as opposed to the confirmed group (1.3 and 1.6 respectively). This is in keeping with a number of studies that have demonstrated that the higher the pre-treatment triglyceride concentration, the lower the mutation detection rate. Futema et al. assessed patients with FH according to the Simon Broome criteria and reported a 60% genetic mutation detection rate in individuals with a pre-treatment triglyceride concentration of 0.4–1.0 mmol/l, compared to 20% in the top quartile (2.16–4.30 mmol/l).^13^ It seems probable, therefore, that a substantial proportion of patients without an identifiable mutation had a Fredrickson type IIb hyperlipoproteinemia, and some others possibly type III or IV; all of which are associated with higher triglyceride concentrations.

A higher proportion of the genetically confirmed patients needed coronary artery bypass graft surgery suggesting there is more multi-vessel disease as opposed to single vessel or less severe disease which can be treated with percutaneous coronary intervention (PCI). Accordingly, there was no difference in PCI requirement between the two groups. There was a higher prevalence of prior myocardial infarction in the genetically confirmed group suggesting a less favourable phenotype.

Our study has limitations in terms of sample size and we acknowledge that it is based on data from a single tertiary cardiac centre which specialises in the care of FH; hence reproducibility of our findings to a wider population may be limited. Although it is difficult to draw too many conclusions from a relatively small sample size, it could be argued that some of these observed differences have arisen because we are often referred patients with genetically confirmed FH who have already developed cardiovascular disease; which may therefore represent a selection bias. Nevertheless, our study provides informative data and, importantly, includes non-fatal, in addition to fatal, cardiovascular events in contrast to the Simon Broome Register which is restricted to fatal events. ^17^

We also recognise that using a modified version of the Simon Broome criteria complicates comparison with previously published studies that have employed the original criteria. We opted to use a modified version in which “family history of myocardial infarction” is replaced with “personal or family history of cardiovascular disease: below age of 50 years in second-degree relative or below age 60 years in first-degree relative”. This is because the conventional criteria potentially overlooks personal premature cardiovascular disease; and the term cardiovascular disease is used in lieu of myocardial infarction as it is more inclusive and may for example include ischaemic strokes which are clinically relevant; which would otherwise be overlooked using the conventional Simon Broome criteria which only includes myocardial infarction.

In accordance with our findings, a recent study evaluated the effect of monogenic mutations and polygenic causes of FH on premature (age <55 years) CVD events in patients with clinically diagnosed FH and demonstrated that a monogenic cause of FH was associated with significantly greater risk of CVD (adjusted hazard ratio: 1.96; 95% confidence interval: 1.24 to 3.12; p=0.004), whereas the risk of CVD in patients with polygenic FH was not significantly different compared with patients in whom no genetic cause of FH was identified.^18^ Furthermore, the presence of an elevated low-density lipoprotein cholesterol (LDL-C) polygenic risk score further increased CVD risk in patients with monogenic FH (adjusted hazard ratio: 3.06; 95% confidence interval: 1.56 to 5.99; p=0.001).^18^ Ideally larger scale prospective studies including cost-effectiveness analysis are required to confirm these findings and conclude whether genetic screening is clinically and financially justified. However, to our knowledge this is one of very few studies to date that seeks to assess whether there is an increased risk of cardiovascular events amongst genetically confirmed FH compared to unconfirmed cases. We have also highlighted differences in baseline characteristics between these two groups which may modify this difference in cardiovascular risk.

## Conclusion

We performed a single tertiary cardiac centre retrospective evaluation of 257 patients with FH and observed a higher prevalence of cardiovascular events in patients with genetically confirmed heterozygous FH compared to patients who were genetically unconfirmed, indicating that genetic testing may help to identify a sub-group with higher cardiovascular risk. Prevalence of prior myocardial infarction and previous coronary artery bypass graft surgery were higher in the context of more significantly raised lipid profiles in the genetically confirmed group suggesting a less favourable phenotype. However, there was a higher prevalence of alternative cardiovascular risk factors in the unconfirmed group including hypertension, co-morbidities, higher age and body mass index of which clinicians need to be aware, which may have modified the difference in cardiovascular risk.

## Acknowledgement

We would like to thank FH specialist nurses Lorraine Priestley-Barnham, Charis Browne, Laura Davis and Cathie Huggett as well as pH Associates for their help and contribution to this work.
